# Monte Carlo and analytic modeling of an Elekta Infinity linac with Agility MLC: Investigating the significance of accurate model parameters for small radiation fields

**DOI:** 10.1002/acm2.12485

**Published:** 2018-11-08

**Authors:** Sara Gholampourkashi, Joanna E. Cygler, Jason Belec, Miro Vujicic, Emily Heath

**Affiliations:** ^1^ Carleton Laboratory for Radiotherapy Physics Carleton University Ottawa ON Canada; ^2^ Department of Physics Carleton University Ottawa ON Canada; ^3^ Department of Medical Physics The Ottawa Hospital Cancer Centre Ottawa ON Canada

**Keywords:** Agility, analytic model, Elekta, Monaco, Monte Carlo, virtual source

## Abstract

**Purpose:**

To explain the deviation observed between measured and Monaco calculated dose profiles for a small field (i.e., alternating open‐closed MLC pattern). A Monte Carlo (MC) model of an Elekta Infinity linac with Agility MLC was created and validated against measurements. In addition, an analytic model which predicts the fluence at the isocenter plane was used to study the impact of multiple beam parameters on the accuracy of dose calculations for small fields.

**Methods:**

A detailed MC model of a 6 MV Elekta Infinity linac with Agility MLC was created in EGSnrc/BEAMnrc and validated against measurements. An analytic model using primary and secondary virtual photon sources was created and benchmarked against the MC simulations and the impact of multiple beam parameters on the accuracy of the model for a small field was investigated. Both models were used to explain discrepancies observed between measured/EGSnrc simulated and Monaco calculated dose profiles for alternating open‐closed MLC leaves.

**Results:**

MC‐simulated dose profiles (PDDs, cross‐ and in‐line profiles, etc.) were found to be in very good agreements with measurements. The best fit for the leaf bank rotation was found to be 9 mrad to model the defocusing of Agility MLC. Moreover, a very good agreement was observed between results from the analytic model and MC simulations for a small field. Modifying the radial size of the incident electron beam in the BEAMnrc model improved the agreement between Monaco and EGSnrc calculated dose profiles by approximately 16% and 30% in the position of maxima and minima, respectively.

**Conclusion:**

Accurate modeling of the full‐width‐half‐maximum (FWHM) of the primary photon source as well as the MLC leaf design (leaf bank rotation, etc.) is essential for accurate calculations of dose delivered by small radiation fields when using virtual source or MC models of the beam.

## 1 INTRODUCTION

Monte Carlo (MC) techniques are accepted to be the most accurate method of dose calculation in radiotherapy and a reliable tool for modeling linear accelerators (linacs).^1^ Creating a reliable dose calculation tool requires accurate and detailed knowledge of the geometry and material of the linac components as well as the characteristics of the incident electron beam through a precise benchmarking of MC model against measurements.^1^, ^2^

Many researchers have studied several linac designs using MC codes to model the geometry of the treatment head and to derive beam parameters for different beam energies.^3^, ^4^, ^5^, ^6^, ^7^, ^8^, ^9^, ^10^, ^11^, ^12^ The methodology adopted by these groups was to create a model of the linac head based on the vendor provided information and to match depth‐dose and dose‐profile curve simulations against measurements to determine the initial beam parameters. In some cases, as reported by Chibani and Ma,^8^ corrections to the information provided by the vendor might be required.

The sensitivity of the linac model to different parameters has been investigated by several groups.^5^, ^6^, ^9^, ^11^, ^13^, ^14^, ^15^ Sheikh‐Bagheri and Rogers^5^, ^6^ studied beam parameters of nine megavoltage photon beams from different manufacturers (Varian, Elekta and Siemens) and concluded that MC simulations of photon beams are highly sensitive to the radial intensity and mean energy of the incident electron beam. They also reported that the accuracy of simulations is sensitive to the primary collimator opening and flattening filter material and density. Chibani and Ma^8^, ^9^ investigated the influence of different parameters of the incident electron beam on Varian photon beams of different energies. In addition to confirming results reported by Sheikh‐Bagheri and Rogers,^5^, ^6^ they showed that large field sizes (e.g., 35 × 35 cm^2^) are quite sensitive to the angular divergence of the electron beam.^9^ Keall et al.^11^ found that MC simulations are sensitive to changes in radial distribution and mean energy of the initial electron beam as well as the target density. Other groups^13^, ^14^, ^15^ confirmed those results and showed that accurate tuning of the incident electron beam parameters is very important to achieve the best match between MC simulations and measurements. Although Bush et al.,^14^ investigated the impact of deviating from Gaussian intensity distribution, the optimal shape of the electron radial intensity profile was confirmed to be Gaussian. This is the shape adopted in all studies that model beam parameters in MC simulations.

An alternative approach of modeling treatment beams is using virtual source models (VSMs). A VSM typically comprises of multiple virtual sources that simulate the contributions from different components of the treatment head. These typically consist of the photons from the target, primary collimator and flattening filter as well as electron contamination.^16^, ^17^, ^18^, ^19^, ^20^, ^21^ The data for particles (e.g., position and direction) generated by each source are derived from the phase space file calculated by MC simulations and scored in a specific plane. Tuning of parameters (e.g., virtual source size, energy fluence, weight of each source) of the VSMs can be achieved by comparison against MC simulations and/or measurements**.** Chabert et al.^20^ created a virtual source model of the Elekta Synergy 6 MV photon beam using phase space data file calculated by the PENELOPE^22^ MC code and scored below the flattening filter. Their VSM model included three virtual sources including a primary source (photons from the target) and two scattered sources (photons from the primary collimator and flattening filter). They implemented their VSM in PENELOPE and investigated the accuracy of dose calculations and portal image prediction with regard to different binning methods to process particle information. Sikora et al.^23^ showed that for field sizes smaller than 2 × 2 cm^2^, precise modeling of the size and contribution of the primary photon source (i.e., photons from the target) is of high importance. They showed that to achieve a good agreement between calculated and measured cross‐ and in‐line profiles for a 0.8 × 0.8 cm^2^ field, the FWHM of the primary photon source needs to be reduced by at least 30% from its original value determined for larger field sizes. In any VSM, all calculations related to virtual sources and resultant photon fluence are according to analytic and mathematical functions describing the source properties. Besides less complexity, another advantage of using VSMs is faster calculation time compared to full MC simulation.

The virtual source model implemented in the Monaco treatment planning system (Elekta AB, Stockholm, Sweden) for Elekta linear accelerators is based on the VSM introduced by Sikora et al.^19^ Their model was initially created for the Elekta Precise SLi linac and includes three virtual sources: (a) primary photon source to model photons generated in the target; (b) secondary photon source to model photons scattered from the primary collimator, flattening filter, anti‐backscatter plate, and the rest of the linac head components; and (c) electron contamination source. All three sources are defined to have a spatial Gaussian distribution. The primary source has a fixed radial distribution and two other sources have energy‐dependent radial distributions. Particle and energy fluence for each source are derived from appropriate phase space data stored during MC simulations. Model parameters (e.g., contribution of each source, source size) are then adjusted by comparing calculated dose in water phantom in Monaco against water measurements for an individual linac. In the Monaco beam model, the MLC as well as the jaws are included and modeled using transmission probability filters. Resulting particles from the model are finally used as input to the x ray voxel Monte Carlo (XVMC)^24^ dose calculation algorithm for dose calculations within the patient.

The relationship between appropriate modeling of the MLC leaf parameters and accurate dose calculations in treatment planning systems has been investigated by several research groups.^25^, ^26^, ^27^ Bedford et al.^25^ verified the performance of the Agility MLC model implemented in the beam model of the Pinnacle^3^ (Philips Radiation Oncology System, Fitchburg, WI, USA) for calculations and delivery of VMAT plans. Kinsella et al.^26^ and Synder et al.^27^ used specifically designed measurements to fine tune model parameters (e.g., leaf transmission, groove width, interleaf leakage) of the Agility MLC model implemented in the Monaco treatment planning system.

Recent years have seen rapid improvements in the techniques of radiation therapy delivery for cancer treatment. More advanced techniques like intensity modulated radiation therapy (IMRT), volumetric modulated radiation therapy (VMAT), and stereotactic body radiation therapy (SBRT) rely on small radiation fields for high precision conformal dose delivery to a target volume while sparing organs at risk (OAR). This introduces important challenges as small fields are associated with greater uncertainty in the accuracy of beam modeling and clinical dosimetry.^28^, ^29^, ^30^, ^31^ These challenges include charged particle disequilibrium, source occlusion and choice of small detectors (e.g., small ion chambers and diodes) to reduce the effect of volume averaging of large detectors.^28^, ^30^, ^31^ A small radiation field is defined as one whose dimensions are comparable to or less than the lateral range of charged particles.^32^ Based on this criterion, for a 6‐MV photon beam, field sizes equal to or less than 3 × 3 cm^2^ are considered to be small.^28^

The focus of this work is to present a detailed MC model of an Elekta Infinity linac with Agility MLC leaves. This MC model was created in EGSnrc/BEAMnrc and benchmarked against appropriate measurements. MLC model parameters were tuned using an alternating open‐closed field which is highly sensitive to model parameters. Comparison of dose profiles obtained from Monaco calculations and measurements/simulations for this same field revealed large discrepancies. Therefore, an analytic model using multiple virtual sources was created to investigate the impact of different beam parameters on the photon fluence at the isocenter plane. Results from the analytic model and EGSnrc simulations are used to explain the aforementioned dose discrepancies between Monaco calculations and measurements.

## 2 METHODS AND MATERIALS

### 2.A Monte Carlo user codes

All MC simulations were performed using EGSnrc^33^ (V4‐2.4.0, National Research Council of Canada, Ottawa, ON, Canada). The BEAMnrc user code^34^ was used to model the Elekta Infinity linear accelerator with Agility MLC. Dose calculations in the water phantom were performed using the DOSXYZnrc user code.^35^ The calculated dose from MC simulations was converted into absolute dose using the following formulation:

(1) $$\begin{array}{cc} D(cGy)= & \frac{{\left(D/\#\;of\;incident\;particles\right)}_{MC\;individual\;simulation}}{{\left(D/\#\;of\;\;incident\;particles\right)}_{MC\;calibration\;simulation}}\\ & \times \frac{1\;cGy}{\mathrm{MU}}\times MU_{\mathrm{del}} \end{array}$$

where, $MU_{\mathrm{del}}$ is the monitor units (MU) delivered by a linear accelerator. In this formula, D/#ofincidentparticles represents the dose scored per number of incident particles in MC simulations. The calibration simulation was performed in water for a square field of 10 × 10 cm^2^ and SSD of 100 cm and dose was scored at a depth of 10 cm.

### 2.B BEAMnrc model of an Elekta Infinity linear accelerator

**Figure 1:**
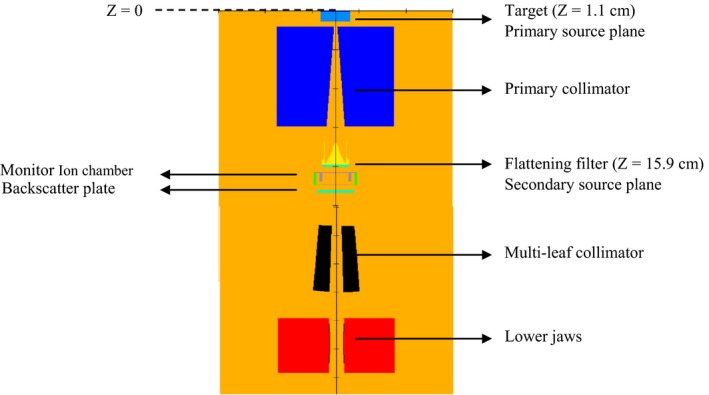
BEAMnrc preview of the Elekta Infinity linac model with Agility MLC showing the various component modules.

A detailed model of an Elekta Infinity linac with Agility MLC was created using BEAMnrc. The linac model was constructed for 6 MV energy, based on the technical data provided by the manufacturer and previously published work.^10^ A geometrical illustration of this model including the patient independent (target, primary collimator, flattening filter, monitor ion chamber, and backscatter plate) and patient dependent (160 leaves, lower jaws) components is shown in Fig. 1.

To model the multi‐leaf collimator and lower jaws, the SYNCMLCE and SYNCMLCQ component modules (CMs) were used, respectively. The “SYNC” versions of these CMs enable synchronization of the motion of the multi‐leaf collimator, gantry and jaws in the linac model, and dose calculation geometry by using a common, randomly generated MU index which lies between 0 and 1, to sample the configuration of the linac components for each particle history. A “SYNC” version of the MLCQ component module was created by modifying this CM to read the MU index generated in the SYNCMLCE CM.

All measurements of output factors, percentage‐depth‐dose (PDD), and cross‐ and in‐line profiles were performed in a water tank (Blue Phantom^2^, IBA Dosimetry, Schwarzenbruck, Germany) on an Elekta Infinity linac operating at a dose rate of 550 MU/min. For output factor measurements, 100 MU was delivered for each field size. Measurements for small field sizes were performed using Exradin A16 ionization chamber (Standard imaging Inc., Middleton, WI, USA) as well as RFD photon diodes (IBA Dosimetry, Schwarzenbruck, Germany). For larger field sizes, CC13 ionization chamber (IBA Dosimetry, Schwarzenbruck, Germany) was used. PDD and profiles were measured using CC13 ionization chamber while buildup and penumbra regions were measured using RFD photon diodes. All measurements were performed in a water tank at an SSD equal to 100 cm. PDD curves were measured for a 5 × 5 cm^2^ field size while output factors as well as cross‐ and in‐line profiles were measured for various small and large field sizes (2 × 2 cm^2^ up to 40 × 40 cm^2^) at 5 cm depth.

**Figure 2:**
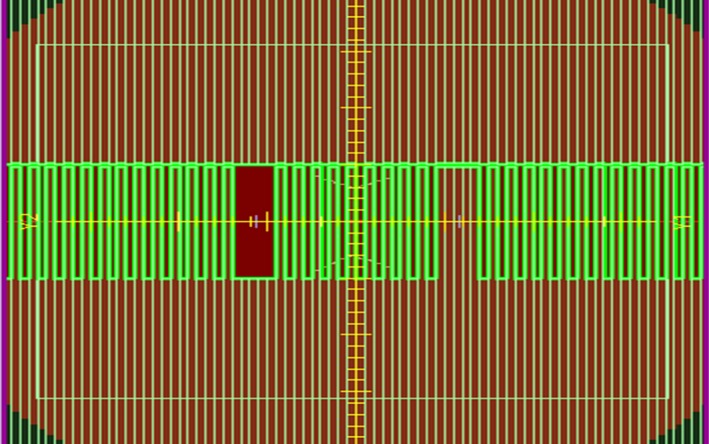
BEV of the fields constructed to evaluate the LBROT value. The fields consist of a small field size (1‐open leaf) to verify leaf bank rotation and one larger field size (5‐open leaves) for dosimetric verification.

One feature of the Agility MLC is defocusing of the leaf bank (i.e., leaf bank rotation) rather than using a tongue and groove to reduce the interleaf leakage. In order to extract the leaf bank rotation (LBROT) value, a field with alternating open‐closed MLC leaf pairs was created in Monaco V.5.11.01 (Elekta AB, Stockholm, Sweden). Dose profiles for this small field size (i.e., 5 mm along the in‐line/leaf bank rotation direction) are highly sensitive to LBROT variations. Two sections with five adjacent leaves open or closed (i.e., 2.5 cm along the in‐line and 10 cm in the cross‐line directions) were included in the pattern as well, to compare dose values for larger field sizes. A beam's eye view (BEV) of the described field is shown in Fig. 2. Dose profiles for this field were measured using a calibrated Gafchromic film (EBT3, Ashland, Wayne, NJ, USA). Point doses were measured using an A1SL ionization chamber (Standard imaging Inc., Middleton, WI, USA) at the center of the 5‐open and 5‐closed leaves sections, 5.75 cm laterally from the isocenter. Dose measurements were performed at SAD of 100 cm at a depth of 5 cm in a 30 × 30 × 10 cm^3^ Solid Water (RMI457 Gammex, WI, USA) phantom.

Source 19^36^ was used as the particle source in BEAMnrc to define the incident electron beam. Source 19 is an elliptical beam source with Gaussian intensity distributions defined in X (cross‐line) and Y (in‐line) directions, with either parallel or angular spread. The energy of the monoenergetic electron beam was determined by matching PDD curves from measurements and MC calculations for a 5 × 5 cm^2^ field. Penumbrae of measured and simulated profiles in both cross‐ and in‐line directions were matched to extract the FWHM value of the electron beam source along both directions independently. Small fields of sizes 2 × 2 cm^2^ and 5 × 5 cm^2^ were chosen because dose profiles of small fields are less sensitive to the mean angular divergence of the initial electron beam.^9^ The mean angular divergence of the electron beam was adjusted by comparing calculated and measured dose profiles of large fields (i.e., 30 × 30 cm^2^ and 40 × 40 cm^2^). The FWHM value was then further tuned by matching measured and simulated relative output factors (ROFs).

As for the LBROT, measured dose profiles for the alternating open‐closed MLC pattern were compared to simulations with varying LBROT values until the best agreement between measurements and simulations was found. The resultant leaf bank rotation causes a translation in the center of the field opening at the isocenter. The appropriate shift, calculated by following equation, was applied to the SYNCMLCE component in the beam model of the linac to account for this translation.

(2) $$shift_{\mathrm{MLC}}=\left(Leaf\;thickness\times \;sin\left(LBROT\right)\right)/2$$

This shift was confirmed by calculating the offset between the center of the open fields of sizes 1 × 5, 5 × 5, and 15 × 15 cm^2^ from MC simulations and measurements. Measurements were performed at SAD equal to 100 cm and 100 MU was delivered to the film at 5 cm depth of the solid water.

The MLC density, composition, and interleaf air gap were first set to the values specified by the manufacturer. These values were then adjusted to find an agreement between calculated and measured leaf transmission values utilizing the field shown in Fig. 2 and according to the explained procedure. Leaf transmission was measured using ion chamber measurements at the position of 5‐open and 5‐closed leaves, respectively. The interleaf air gap was then tweaked until the best agreement between measurements and simulations was found for the single leaf openings in the same field. Once interleaf air gap value is modified, the leaf transmission changes. As a consequence, the MLC density and composition as well as the interleaf air gap were tweaked again to match the measured leaf transmission value. The process was then repeated until no further improvements were observed.

For all simulations, the photon cutoff energy (PCUT) and electron cutoff energy (ECUT) were set to 0.01 and 0.7 MeV, respectively, and the electron range rejection was set to 2 MeV. Bremsstrahlung cross‐section enhancement was turned on and the directional bremsstrahlung photon splitting algorithm was used.

### 2.C Analytic model of the Elekta Infinity linac

An analytic model of the Elekta Infinity linac was created in Python as a simple and fast method to assist with understanding the impact of different input parameters on the photon fluence at the isocenter plane from small radiation fields. The model parameters were tuned by comparison against MC simulations using the validated BEAMnrc model of the Elekta Infinity linac as described in Section 2.B. The analytic model consists of two virtual photon sources (primary and secondary) as well as a model of the Agility MLC leaves. In order to extract parameters for the analytic model, the BEAMnrc user code was modified to score photon fluence and position as well as other photon characteristics (energy and angular distributions) at different planes of the MC model of the linac. This helped to avoid saving large phase space files that contain large number of particles to extract the needed information.

The primary photon source was used to model the bremsstrahlung photons generated in the target. In the MC linac model, photons were scored below the flattening filter, *Z* = 15.9 cm from the reference plane (*Z* = 0 cm), and back projected to the distal side of the target at *Z* = 1.1 cm. These planes are shown on Fig. 1. Only photons whose projected trajectories intersected the isocenter plane (*Z* = 100 cm) within the in‐line distance of ±10 mm from the isocenter were included in the primary source. These limits were chosen based on the small field size (5 × mm^2^ at isocenter) and the fact that bremsstrahlung photons coming out of the target are fairly forward‐peaked. The resultant photon fluence was found to fit a Gaussian distribution with a FWHM = 1.05 mm.

**Figure 3:**
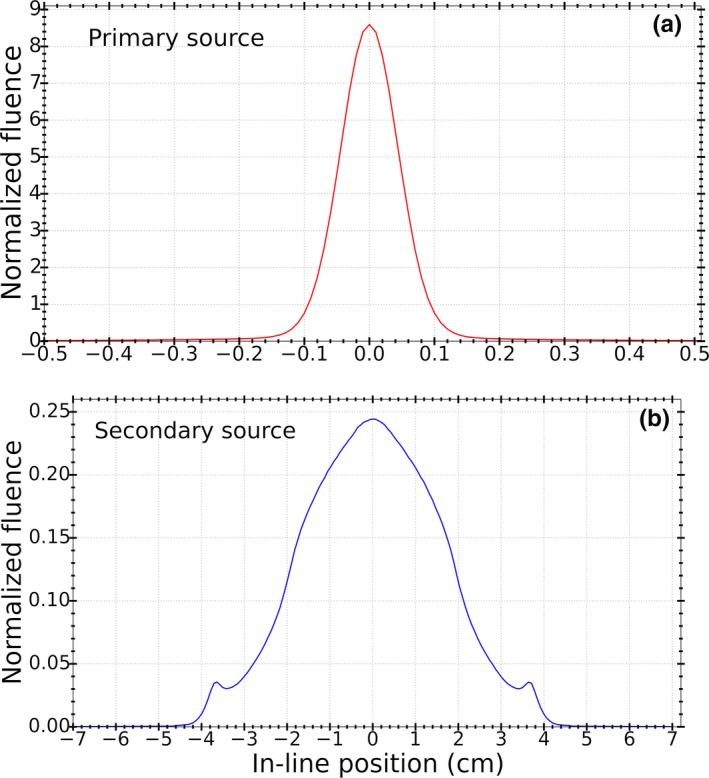
(a) Primary and (b) secondary virtual photon sources used in the analytic model. The primary and secondary photon sources are placed at *Z* = 1.1 cm and *Z* = 15.9 cm from the reference plane (*Z* = 0 cm), respectively.

The secondary photon source was created to model the scattered photons coming from the primary collimator and flattening filter and was located at the distal plane of the flattening filter at *Z* = 15.9 cm. A photon elimination criterion of ±7 cm in the in‐line distance was adopted to score photons for the secondary source. Both virtual sources are shown in Fig. 3.

**Figure 4:**
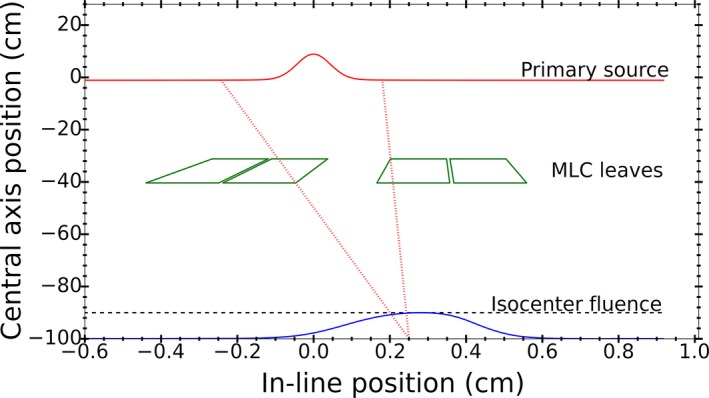
Ray diagram illustrating the photon fluence calculation process of the analytic model. The fluence at each point along the in‐line position on the isocenter plane is the integral of the source. The source boundaries are shown by the photon rays tracing from the isocenter to the source plane.

In order to calculate fluence profiles at the isocenter plane, a ray tracing algorithm was used to trace the trajectories of photons from both virtual sources through the MLC. The geometry of the Agility MLC leaves, including the leaf bank rotation, was extracted from MC simulations and adopted into the analytic model. The source boundaries for fluence calculation comprise the points of non‐zero fluence plus an additional 5 mm margin on either side boundaries. Attenuation of photons in the MLC leaves was modeled by exponential attenuation. Attenuation coefficient data for the leaf material composition was obtained from NIST XCOM database using the average photon energy distribution obtained from MC simulations for each virtual source. The contribution of each virtual source to the resultant photon fluence at the isocenter plane was derived from the ratio of the number of photons from each source to the total number of photons at the isocenter plane from MC simulations. Figure 4 illustrates the ray diagram for the analytic model.

### 2.D Impact of analytic model parameters on the fluence at the isocenter plane

The impact of the analytic model parameters on the photon fluence at the isocenter plane for a single open leaf field was studied. LBROT, the size of the primary photon source, angular and energy distributions and attenuation in the MLC leaves were modified and their impact on the fluence was investigated. Moreover, since the MLC model in Monaco also includes a tongue and groove design (the Monaco beam model parameters include a tongue and groove width), the impact of parameter tongue and groove on the fluence was studied using the analytic model. Finally, the impact of exclusion of the secondary photon source was investigated. Our expectation was that the secondary photon source would have limited impact due to the fact that head scatter decreases significantly as a result of source occlusion at small field sizes.^37^ Fluence profile changes were quantified in terms of maximum photon fluence, integral of the fluence profile and distance‐to‐agreement (DTA) at the penumbra region (50% of the maximum fluence).

### 2.E Comparison of Monaco and EGSnrc dose calculations

Dose profiles from measurements, Monaco and EGSnrc calculations were compared for the alternating open‐closed MLC pattern field described in Section 2.B. The voxelized geometry for dose calculation was defined according to the geometry and material of the solid water phantom used for dose measurements in Section 2.B. Voxel sizes were defined to be 1 × 1 × 1 mm^3^ to match the resolution of film measurements. For dose calculations using DOSXYZnrc, the linac model described in Section 2.B was used as a particle source (Source 9)^35^ thus eliminating the need to store a separate phase space file. A photon splitting value of 40 and other transport parameters as described in Section 2.B were used for all DOSXYZnrc simulations. Dose was calculated at 5 cm depth of the voxelized geometry phantom and same beam settings as explained in Section 2.B. Dose calculations were performed with 2 × 10^9^ histories to achieve a mean relative statistical uncertainty^38^ of 0.5% over all voxels with doses greater than 50% of the maximum dose.

Dose calculations in Monaco were performed using the XVMC^24^ algorithm. A 1 mm dose calculation grid with a statistical uncertainty of 1% was used for dose calculations in Monaco. Details of the beam model for the Elekta Infinity linac and Agility MLC in Monaco were used to change source parameters (FWHM of the incident electron source) and geometry of the MLC leaves (inclusion of tongue and groove with groove width of 0.4 mm) in the BEAMnrc model of the linac.

### 2.F Comparison metrics

For comparison purposes, point dose differences between EGSnrc and Monaco calculated dose values were determined. These difference comparisons were quoted as the percentage of the EGSnrc simulated dose values at the point of comparison. Similar comparisons were performed between Monaco/EGSnrc calculations and measurements with the difference comparisons defined as the percentage of the measured dose values at the point of comparison. To compare dose profiles from EGSnrc simulations and measurements, a one‐dimensional (1D) global gamma analysis^39^ with various criteria (i.e., 1%/1 mm and 2%/1 mm) was utilized with measurement dose used as the reference.

## 3 RESULTS

### 3.A BEAMnrc model of Elekta Infinity linear accelerator

**Figure 5:**
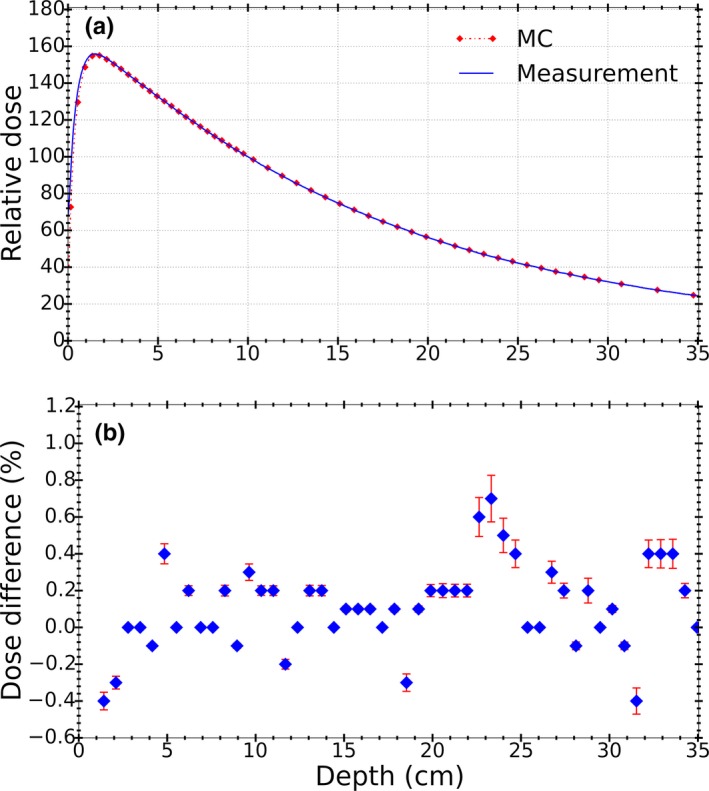
(a) Comparison of the measured and calculated PDD curves for a 5 × 5 cm^2^ field at SSD = 100 cm normalized at 10 cm depth, (b) Percent dose differences for calculated point doses against measurements. Error bars represent statistical uncertainty from MC simulations (0.1%).

**Figure 6:**
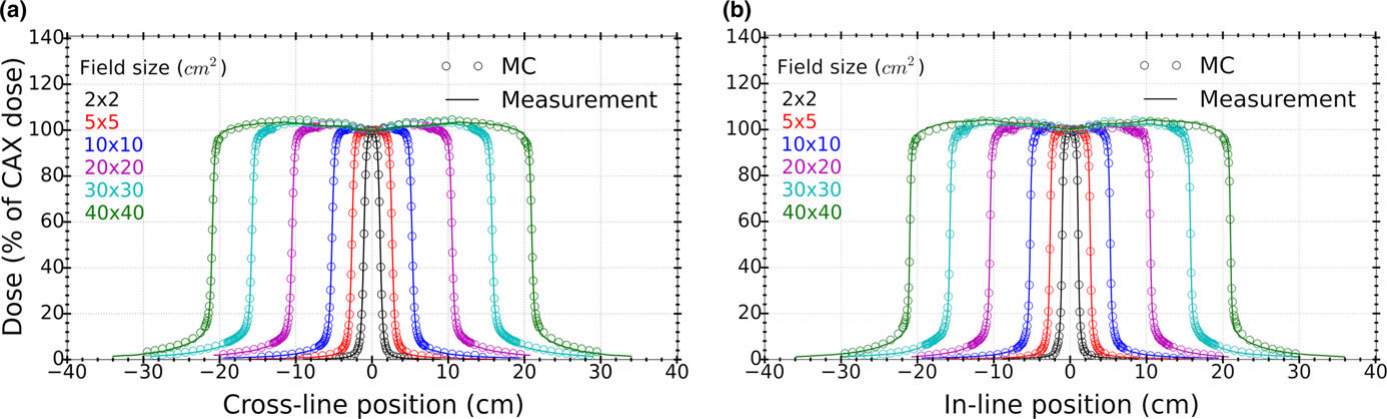
Comparison of the measured and calculated (a) cross‐line and (b) in‐line profiles for various field sizes at 5 cm depth and 100 cm SSD.

**Figure 7:**
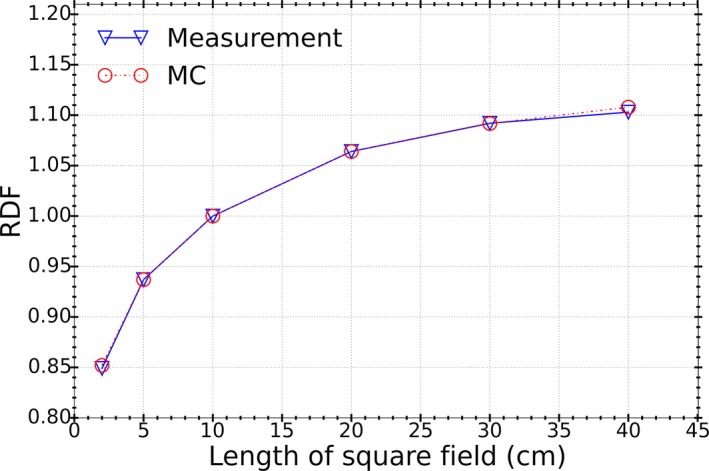
Comparison of the measured and calculated relative output factors for various field sizes at 5 cm depth and 100 cm SSD.

Figures 5, 6, 7 show the comparison of commissioning data and MC calculations for the Elekta Infinity linac. Excellent agreement was observed between measured and simulated PDD curves as illustrated in Fig. 5(a). All dose points past the buildup region passed a 1%/1 mm gamma comparison. Also, over 90% of dose points from MC simulations were found to be within 0.5% of measurements as shown in Fig. 5(b).

The cross‐line and in‐line profiles [Figs. 6(a) and 6(b)] also showed good agreement between measurements and MC calculations. For cross‐line profiles, all points from MC calculations passed a 2%/1 mm gamma analysis when compared against measurements. For the same criteria applied to in‐line profiles, passing rates of 100% and over 95% were observed for field sizes smaller than or equal to 20 × 20 cm^2^ and larger than 20 × 20 cm^2^, respectively.

Average DTA (left and right) values between MC calculated and measured data, in penumbra region (50% of the relative dose), for field sizes from 2 × 2 cm^2^ to 40 × 40 cm^2^ were found to be better than 0.1 mm.

Figure 7 shows a comparison of measured and MC calculated ROFs for several field sizes. The agreement was found to be very good for all field sizes, with the largest discrepancy of less than 0.5% for the 40 × 40 cm^2^ field size.

**Figure 8:**
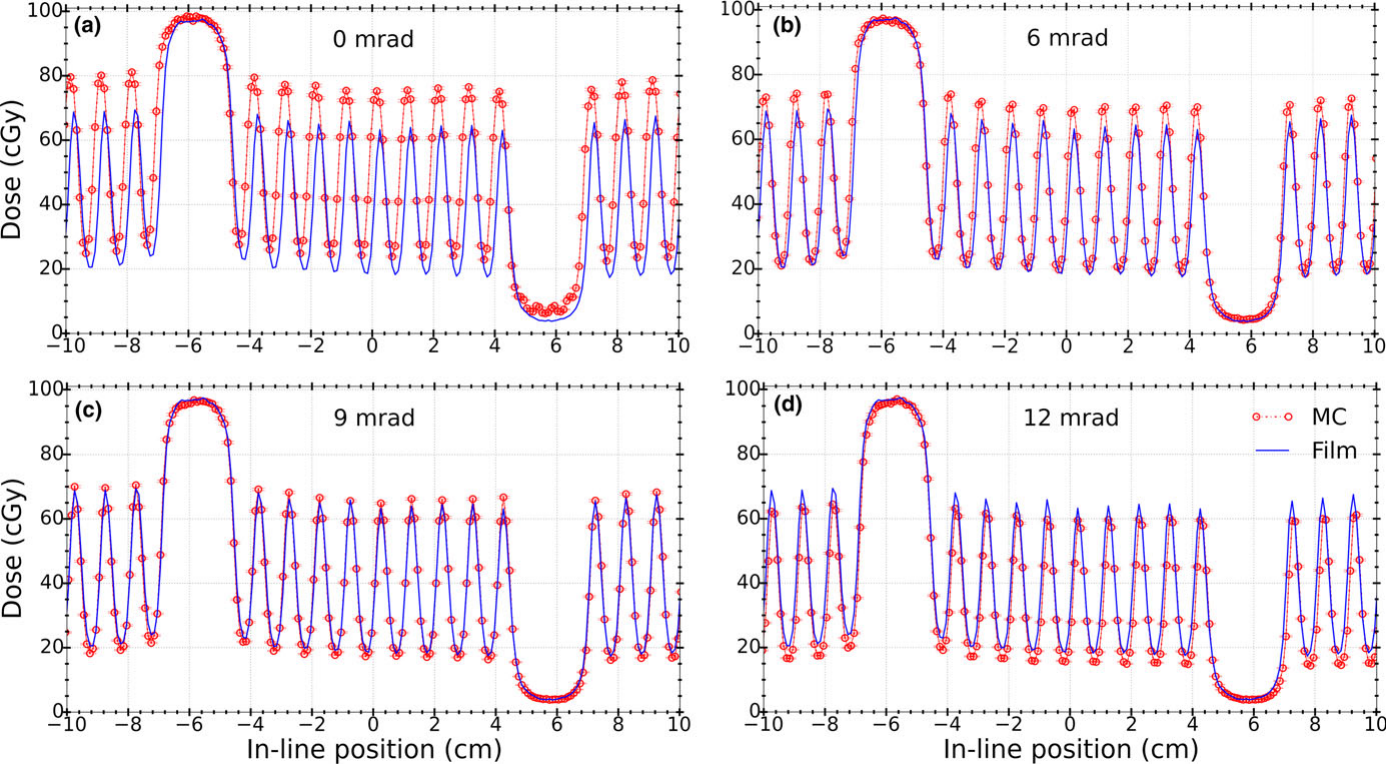
Comparison of dose profiles between EBT3 film measurements (solid) and MC simulations (dotted) for the field shown in Fig. 2 for LBROT values of (a) 0, (b) 6, (c) 9, and (d) 12 mrad. The best fit parameter was found to be LBROT = 9 mrad.

Figure 8 shows the measured and simulated dose profiles for the alternating leaf field for LBROT values of 0, 6, 9, and 12 mrad. Percentage dose differences at the maxima (corresponding to 1‐open leaf) and minima (corresponding to 1‐closed leaf) as well as DTA values at the penumbra region (50% of the maximum dose) are shown in Table 1.

**Table 1:** Mean percentage dose differences at the maxima and minima as well as average DTA (left and right) values at penumbra region from film measurements and MC simulations for different LBROT values. Uncertainties are statistical uncertainties associated with dose values at different maxima and minima from the profiles

LBROT (mrad)	Dose difference (MC/Film) %		Average DTA (mm)
	Maxima (1‐open leaf)	Minima (1‐closed leaf)	
0	17.5 ± 1.5	31.7 ± 3.0	3.0
6	8.7 ± 1.3	9.2 ± 1.8	1.0
9	2.2 ± 1.1	−5.1 ± 1.3	0.2
12	−7.1 ± 1.0	−14.1 ± 1.0	1.0

From the data presented in Fig. 8 and Table 1, the impact of increasing leaf bank rotation on the isocenter dose profile can be seen. Also, it can be observed that the best agreement corresponds to MC simulations with LBROT value of 9 mrad. The resultant translations in the MLC leaf bank, as derived from measurements and MC simulations and derived by suggested formulations, were 0.42 and 0.41 mm, respectively.

**Figure 9:**
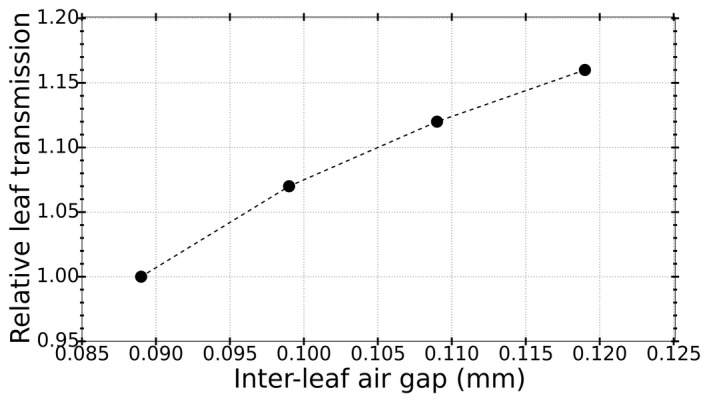
Variation of leaf transmission by increasing the interleaf air gap. All transmission values are normalized to the transmission corresponding to the nominal interleaf air gap.

Using ion chamber measurements at the position of 5‐open and 5‐closed leaves, the average leaf transmission was measured to be (4.3 ± 0.1)%. From MC simulations, transmission was calculated to be (4.1 ± 0.1)%. It was observed that a decrease of 1.1% in the density of leaves (i.e., 18.7 to 18.5 g/cm^3^) increases leaf transmission by 2.5%. Impact of interleaf air gap on leaf transmission for LBRTO value of 9 mrad is shown in Fig. 9. The nominal air gap was calculated to be 0.089 mm.

From this plot, we can see that as the interleaf air gap increases by 0.001 mm, the leaf transmission also increases by approximately 6.0%.

Parameters of the Elekta Infinity linac model that were derived based on the above analysis are shown in Table 2.

**Table 2:** Derived parameters of the Infinity linac model with their uncertainties

Parameter	Value
Electron beam energy	6.6 ± 0.1 MeV
Beam width (cross‐line)	2.1 ± 0.1 mm
Beam width (in‐line)	1.0 ± 0.1 mm
Angular divergence	1.35 ± 0.20 deg
Leaf bank rotation angle (LBROT)	9.0 ± 1.0 mrad
Leaf material density	18.5 g/cm^3^

The leaf composition (i.e., tungsten alloy) was modified from the manufacturer provided values according to Table 3.

**Table 3:** The composition of the leaf as provided by manufacturer and adjusted in the MC beam model

Material	Composition percentage	
	Manufacturer	MC
Tungsten (W)	95%	96%
Nickel (Ni)	3.75%	3%
Iron (Fe)	1.25%	1%

### 3.B Analytic model of the Elekta Infinity linac

**Figure 10:**
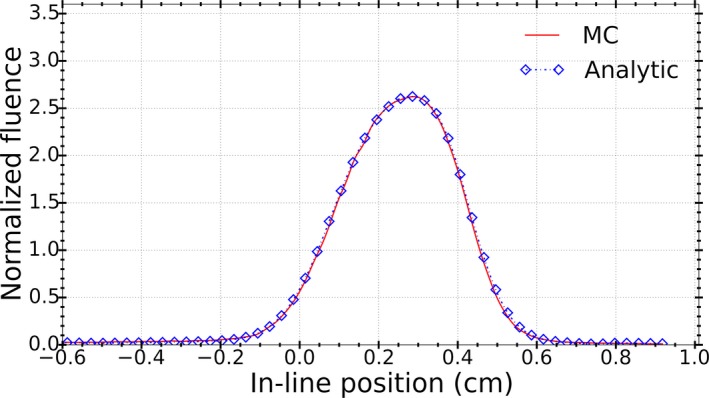
Photon fluence at the isocenter plane from MC simulations and analytic model calculations for LBROT = 9 mrad. Fluence curves are normalized to their integral so that the integral of the resultant curve is equal to 1.

Figure 10 shows a comparison of photon fluence profiles at the isocenter plane from the analytic and MC (BEAMnrc) linac models. Agreement of better than 1% was observed at the position of maximum fluence. Also, the average DTA was found to be 0.04 mm at the penumbra region (50% of the maximum dose).

### 3.C Impact of analytic model parameters on the fluence at the isocenter plane

**Figure 11:**
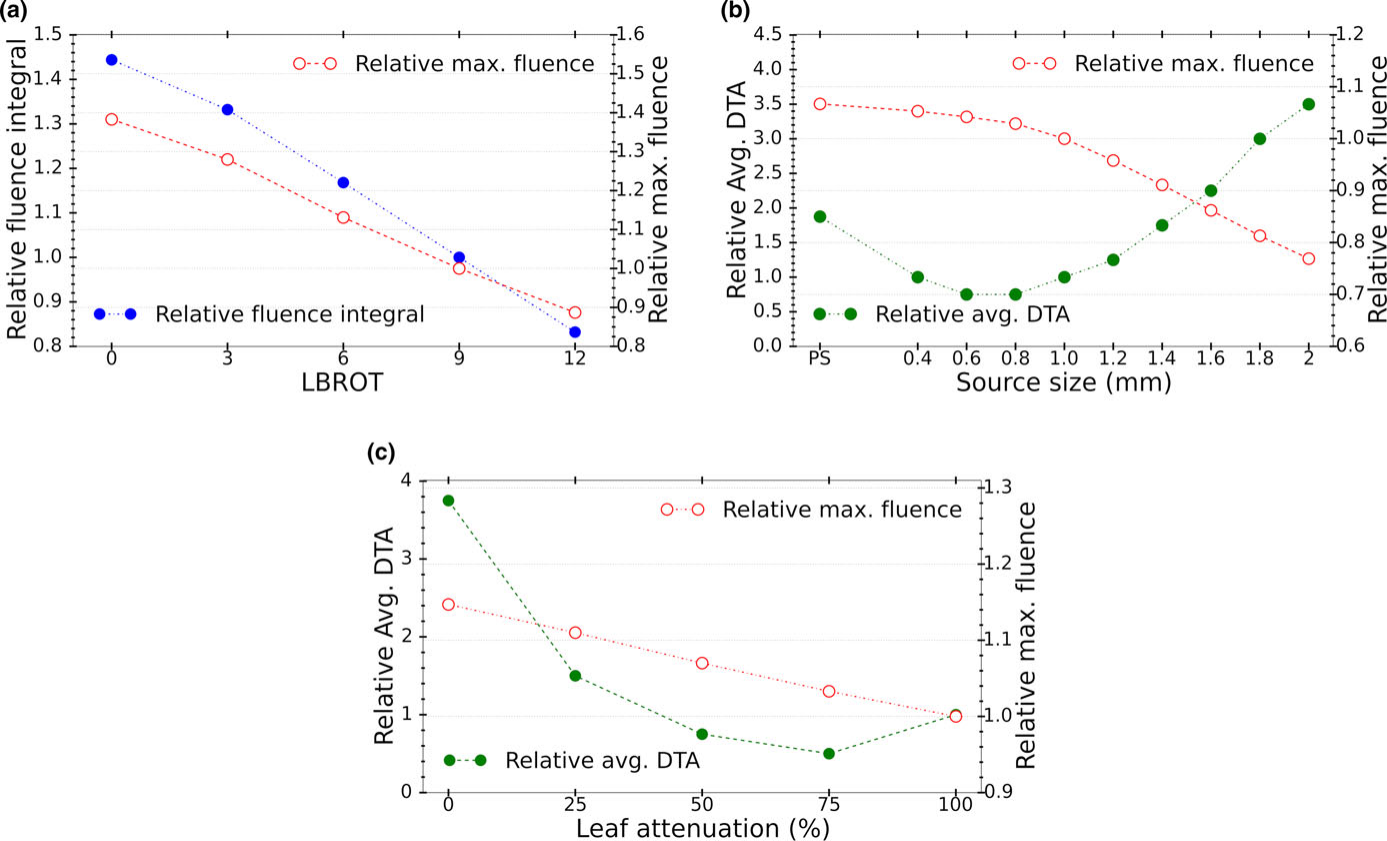
Impact of changes in (a) leaf bank rotation (normalized to nominal value of leaf bank rotation or LBROT = 9 mrad), (b) leaf attenuation (normalized to full MLC leaf attenuation), and (c) primary source size (normalized to nominal source size of 1 mm) on the relative fluence integral, maximum fluence, and average DTA of the fluence in analytic model.

The impact of modifying the analytic model parameters on the fluence at the isocenter plane, as described in Section 2.D, is illustrated in Fig. 11. From Fig. 11(a), we can see that change in the maximum fluence due to increasing leaf bank rotation follows the same trend as in Fig. 8 and Table 1. Due to the source occlusion, the maximum fluence drops from approximately 40% higher to 10% lower than the fluence at the nominal LBROT (9 mrad) as leaf bank rotation increases from 0 to 12 mrad. Ignoring leaf attenuation (100% leaf transmission), as illustrated in Fig. 11(b), increases the maximum fluence by over 12% and average DTA by almost four times (up to 0.16 mm) compared to the scenario where attenuation by the MLC leaves is considered. It can be observed from Fig. 11(c) that when the primary source is changed from a point source to a source with radius of 2 mm the maximum fluence decreases by approximately 25% due to source occlusion. Also, the average DTA worsens once the source size deviates from the nominal value of 1 mm.

Modeling the energy distribution of the primary and secondary photon sources with mean photon energies of 1.6 and 0.7 MV instead of using polyenergetic sources caused a negligible fluence change of approximately 0.1%. However, a decrease of 0.2 MV in the mean energy of the primary photon source decreased the fluence integral by 1.1% due to the increase in the leaf attenuation. Using a uniform angular distribution as opposed to accurately modeling the angular distribution of the photons and its variation across the beam decreased the fluence by approximately 3% and changed the DTA to 0.05 mm. Modeling the tongue and groove in the MLC leaves caused the fluence to increase by over 2% and average DTA by 0.01 mm. Exclusion of the secondary photon source reduced the fluence by less than 1% and caused almost no change in the average DTA. This result was expected as described previously in Section 2.D.

### 3.D Comparison of Monaco and EGSnrc dose calculations

**Figure 12:**
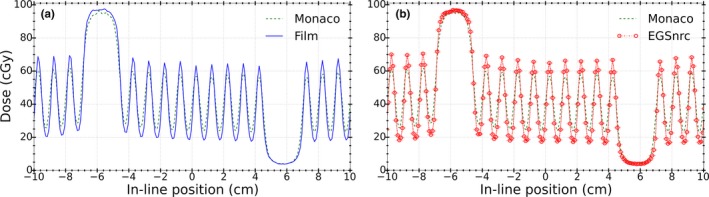
Comparison of Monaco calculations against (a) EBT3 film measurements and (b) EGSnrc simulations for the field shown in Fig. 2.

Comparisons of dose profiles of the field shown in Fig. 2 for Monaco calculations against film measurements and MC simulations using EGSnrc are shown in Figs. 12(a) and 12(b), respectively.

Table 4 shows percentage dose differences and DTA values as similarly reported in Table 1.

**Table 4:** Mean percentage dose differences at the maxima and minima as well as average DTA (left and right) values at penumbra region from Monaco calculations against film measurements and EGSnrc simulations. Uncertainties are statistical uncertainties associated to dose values at different maxima and minima from the profiles

LBROT (mrad)	Dose difference %		Average DTA (mm)
	Maxima (1‐open leaf)	Minima (1‐closed leaf)	
Monaco/Film	−11.3 ± 0.8	33.7 ± 2.9	0.4
Monaco/EGSnrc MC	−13.2 ± 1.0	39.5 ± 1.5	0.5

**Figure 13:**
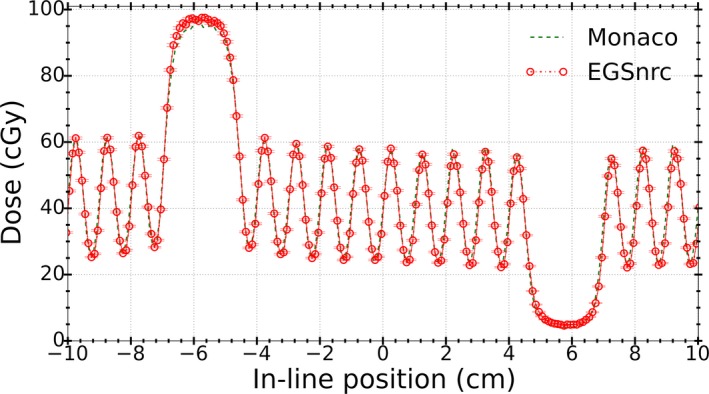
Comparison between Monaco calculations and EGSnrc simulations with modified parameters: FWHM = 2 mm in the in‐line position and inclusion of tongue and groove. Dose differences were reduced to approximately 1% at the maxima and 2% at the minima.

To understand the observed differences, results of the analytic model from Sections 3.B and 3.C were used to modify the parameters in the BEAMnrc model of the linac to find a match between EGSnrc and Monaco calculations. First, the incident electron beam size (FWHM) along the in‐line direction was changed from 1 to 2 mm. Next, the MLC leaf geometry was modified to include the tongue and groove as described in Section 2.E. The resultant dose profile from EGSnrc simulations is compared with Monaco calculations in Fig. 13. It can be observed that implemented modifications in the MC model of the Elekta Infinity linac improved the agreement level between dose profiles from EGSnrc simulations and Monaco calculations.

Table 5 shows the percentage dose differences and DTA values from EGSnrc simulations with modified beam width in the in‐line position as well as inclusion of tongue and groove in the MLC model.

**Table 5:** Effect of varying model parameters in EGSnrc simulations on mean percentage differences of dose at the maxima and minima, as well as average DTA (left and right) values at penumbra regions from Monaco calculations against EGSnrc simulations. Changes were made to increase the in‐line beam width to 2 mm as well as including tongue and groove to the MLC model. Uncertainties are statistical uncertainties associated with dose values at different maxima and minima from the profiles

Parameters modified	Dose difference (Monaco/EGSnrc MC) %		Average DTA (mm)
	Maxima (1‐open leaf)	Minima (1‐closed leaf)	
FWHM _In‐line_ = 2 mm	2.7 ± 1.0	8.0 ± 1.0	0.3
FWHM _In‐line_ = 2 mm + tongue and groove	−0.4 ± 0.5	1.6 ± 0.8	0.2

## 4 DISCUSSION

Benchmarking of the MC model of a 6‐MV Elekta Infinity linac using the method introduced by Almberg et al.^10^ is presented in this study. PDD curves for a 5 × 5 cm^2^ field as well as cross‐ and in‐line profile measurements of different field sizes were used to derive the mean energy and radial intensity (FWHM) of the incident electron beam, respectively. Almberg et al.^10^ used film measurements of the penumbra and buildup regions to take advantage of the energy independent film response. In this work, similar measurements were performed using diodes combined with ion chamber to complement diode measurements and to account for the energy dependence of the diodes in large field sizes. Further adjustment of the FWHM of the radial intensity profile was performed using ROFs. The ROFs of small fields (e.g., 2 × 2 cm^2^) were measured using small volume ion chamber and photon diodes. The angular distribution of the electron beam was determined from profile measurements of large field sizes. A very good agreement was found between MC calculated and measured curves for all PDD, profiles and output factor measurements. A passing rate of 100% was observed when comparing simulated PDD curves against measurements using a 1%/1 mm gamma criteria. As for cross‐ and in‐line profiles, all simulated dose points passed a 2%/1 mm gamma comparison against measurements for field sizes smaller than or equal to 20 × 20 cm^2^. The passing rate for larger field sizes was better than 95%. For ROFs, worst agreement was less than 0.5% for the 40 × 40 cm^2^ field size.

The beam modulation system of the Infinity linac is the Agility MLC comprising 160 leaves with projected leaf width of 5 mm at the isocenter. The choice of an alternating open‐closed leaves field to derive the leaf bank rotation parameter (LBROT) enabled us to apply a small field size and find the optimal value of the leaf bank rotation. An LBROT value of 9 mrad was found to give the smallest dose differences (maxima and minima) and DTA values between MC simulations and film measurements. The optimal interleaf air gap for this leaf geometry was found to be approximately 0.09 mm. The average measured and calculated leaf transmissions were found to agree within 5% of each other. Occasionally, appropriate corrections to vendor provided information about the material and density of different components of the linac head are required.^8^ In our study, the composition and density of the leaves, which are made of a tungsten alloy, were adjusted in the MC model for better agreement with measurements. The fitted values of the MLC leaf composition were within 1% of the values provided by the manufacturer and density was higher by approximately 3%.

A virtual source model of our Elekta linac with Agility MLC was successfully adopted to analytically calculate the photon fluence at the isocenter plane from a small field. The model comprises only two photon sources. The contribution of contamination electrons to the resultant fluence at the isocenter plane was neglected because it was less than 1%. The model used a simple ray tracing and exponential attenuation relationship to model the impact of the MLC leaves. Despite these approximations, our analytic model provided a simple and quick, yet reliable method, to investigate the sensitivity of the fluence to linac model parameters and to explain the disagreements between film measurements and Monaco calculations. For example, it was observed that the fluence at the isocenter plane is highly sensitive to the size of the primary photon source. Also, the integral fluence was shown to be quite sensitive to the change in the mean energy of the primary source. On the other hand, the fluence was minimally sensitive to using mean energy values for the photon sources rather than a spatial energy distribution. These results are in agreement with findings from groups who studied sensitivity of the MC model parameters to the characteristics of the incident electron beam.^5^, ^6^, ^9^, ^11^, ^13^, ^14^, ^15^ Due to the fact that the secondary photon source only represents the scattered photons, the fluence showed to have negligible sensitivity to excluding this source or changing its parameters (e.g., mean energy). However, the contribution of the secondary photon source could become more important for larger field sizes compared to the ones investigated in this study.

Regarding the impact of the leaf bank rotation, the change in the calculated fluence follows the same trend as the dose differences at the maxima in the dose profile of the alternating field as presented in Table 1. Increasing the leaf bank rotation causes a decrease in the fluence due to increased occlusion of the source. Inappropriate modeling of the leaf transmission (e.g., leaf density, attenuation coefficient and leaf thickness) can also affect the fluence at the isocenter plane. However, the sensitivity of the fluence to this parameter was not found to be large since a 25% decrease in the leaf attenuation causes 4% error in the fluence. Thus, we can see that although it is important to properly model the transmission of the leaves, error of a few percent in transmission parameters is not an important source of error in the fluence calculated at the isocenter plane. This result was similar to the findings from MC simulations with modified leaf density, as explained earlier. In the analytic model, the path length of the rays traversing the MLC leaves was defined according to their sampled trajectory. A change of less than 0.1% in the fluence was observed if the rays were assumed to travel exclusively along the direction parallel to the beam axis (as is used in Monaco beam model) rather than the accurate oblique path. This was predictable considering small trajectory angles of the bremsstrahlung photons of the target (i.e., primary photon source) and small contribution of the scatter photons from the primary collimator and flattening filter (i.e., secondary source).

Results of the analytic model calculations confirmed the findings previously shown by Sikora et al.^23^ and showed the importance of accurate modeling of the primary photon source size for the small fields. The change in the size of the primary photon source improved the agreement between Monaco and EGSnrc calculated doses by over 10% and 30% at maxima and minima, respectively. Another important parameter in photon fluence calculations for small field sizes is the modeling of the multi‐leaf collimator. According to the MLC geometry parameters from the Monaco beam model, in addition to the leaf bank rotation, a tongue and groove design with a groove width of 0.4 mm was implemented in the MLC model. To study the impact of this tongue and groove design, the leaf geometry in our BEAMnrc model was modified using the tongue and groove width specifications in the MLC geometry file in Monaco. It was found that addition of tongue and groove to the current model of MLC leaves in the BEAMnrc model improved the agreement at both maxima and minima by about 3% and 6%, respectively, compared to the initial change in the source size (i.e., using a source size with FWHM = 2 mm). This is due to the insertion of groove and as a result less occlusion of the source and higher number of photons reaching the isocenter. Similar results were observed when using a leaf bank rotation of 8 mrad (no added tongue and groove) rather than 9 mrad which results in less source occlusion as well.

From these findings, we can conclude that the observed disagreements between Monaco and measured/EGSnrc calculated dose profiles [Figs. 12(a) and 12(b)] could be associated mainly to the size of the primary source as modeled in the beam model implemented in Monaco. Comparison of EGSnrc simulations using the same focal spot size as in Monaco (i.e., FWHM = 2 mm) against Monaco calculations (Fig. 13) confirmed this conclusion as the dose calculated by EGSnrc dropped by approximately 16% at the maxima or position of 1‐open leaf. Also, since the entire source cannot be viewed from the center of the field at the isocenter (i.e., source occlusion), the penumbra region widens, which results in the reduction of the DTA between EGSnrc and Monaco calculated dose profiles and therefore better agreement. The superposition of widened penumbrae from adjacent open leaves results in a 30% dose increase at the position of 1‐closed leaf or minima of the dose profile. Overall, changing the source size from 1 to 2 mm and insertion of tongue and groove changed the result of EGSnrc simulations to be closer to the calculations obtained from Monaco.

## 5 CONCLUSIONS

Proper values for parameters in the MC model of a linear accelerator head play an important role in accurate dose calculations. The same principle applies when using a virtual source model to analytically calculate the photon fluence resultant from a treatment head. Moreover, with advancements in radiation delivery techniques, using small fields has become inevitable in radiotherapy. This introduces more complexity to fine tuning model parameters of MC or analytic source models (i.e., beam and collimation parameters) due to the challenges associated with small fields.

In this paper, we demonstrated the detailed MC modeling of a 6 MV Elekta Infinity linac with Agility MLC leaves that was validated against measurements. Also, we demonstrated possibility of using a simple analytic model as a quick method to study the sensitivity of different model parameters of a linac when delivering treatments with small field sizes. One important result that was studied using the analytic model and confirmed by MC simulations and measurements was the importance of adjustment of primary photon source size for small fields. Another important result obtained in this study was that modeling of the MLC leaf design (i.e., leaf bank rotation and/or tongue and groove) is essential for accurate simulation of delivered dose (maximum dose and penumbrae). Results from this study helped us explain the discrepancies observed between dose calculations obtained from Monaco treatment planning system and film measurements/EGSnrc simulations of the Elekta Infiniy linac.

In conclusion, simulation of advanced techniques such as IMRT, VMAT, and SBRT that comprise small fields requires a realistic MLC model as well as adjusted size of the primary photon source in the virtual source model used in treatment planning systems. Results of this study could be valuable to cancer centers that use the Elekta Infinity linac to help ensure accurate dose calculations for the above‐mentioned treatment techniques.

## CONFLICTS OF INTEREST

The authors have no relevant conflicts of interest to disclose.
